# Preparing for future outbreaks in Ghana: An overview of current COVID-19, monkeypox, and Marburg disease outbreaks

**DOI:** 10.34172/hpp.2023.25

**Published:** 2023-09-11

**Authors:** Isaac Owusu, Collins Adu, Richard Gyan Aboagye, Rebecca Ann Mpangah, Gideon K. Acheampong, Ernest Akyereko, Emmanuel Osei Bonsu, Prince Peprah

**Affiliations:** ^1^Ghana Health Service, Headquarters, Accra, Ghana; ^2^College of Public Health, Medical and Veterinary Sciences, James Cook University, Townsville, Queensland 4811, Australia; ^3^Fred Newton Binka School of Public Health, University of Health, and Allied Sciences, Hohoe, Ghana; ^4^Faculty of Geo-Information Science and Earth Observation (ITC), University of Twente, Enschede, The Netherlands; ^5^Department of Epidemiology and Biostatistics, Kwame Nkrumah University of Science and Technology, Kumasi, Ghana; ^6^Centre for Primary Health Care and Equity/Social Policy Research Centre, University of New South Wales, Sydney, Australia

**Keywords:** Marburg virus, Monkeypox virus, COVID-19, Ghana, Outbreak response

## Abstract

Amidst the ongoing COVID-19 pandemic, Ghana is currently grappling with simultaneous outbreaks of Marburg virus disease and human monkeypox virus. The coexistence of these outbreaks emphasizes the imperative for a collaborative and global approach to enhance surveillance and expedite case detection. While Ghana has made efforts to respond to these outbreaks, this paper outlines the lessons learned and proposes recommendations in this regard. It is crucial to intensify response efforts at the local, regional, and national levels to effectively contain the spread of these infectious diseases. Therefore, this paper suggests prioritizing the following recommendations as crucial for assisting Ghana in adequately preparing for future outbreaks and safeguarding global public health: strengthening surveillance system through digitization, rapid and effective response; risk communication and community engagement; healthcare system readiness; and research and collaboration. Also, prioritizing building healthy public policies and developing personal skills of health personnel across the country is key for future outbreak response.

## Introduction

 Marburg disease poses a significant global health threat, with recurrent outbreaks occurring primarily in Africa.^1-5^ The viral disease is a member of the Filoviridae family which is known for its highly virulent and deadly haemorrhagic fever, exhibiting a significant case fatality rate in Sub-Saharan Africa.^6-9^ Previous epidemics and sporadic cases of Marburg virus disease (MVD) have occurred in Kenya, Angola, Uganda, Democratic Republic of Congo and South Africa.^6,10^

 A severe disease with a high death rate is caused by MVD, which spreads through direct contact with nonhuman primates and other mammals that are infected.^8,11,12^ The Marburg virus can spread to humans when they touch the bodily fluids, surfaces, or objects that are contaminated by infected fruit bats, which are the natural hosts of the virus.^13^ The onset of the illness is characterized by malaise, severe headache, and high fever, with many patients experiencing severe haemorrhagic symptoms within seven days.^14-16^ The death rates in previous outbreaks have varied from 24% to 88%, depending on the type of virus and the level of care for the patients.^16^ Currently, there are no approved treatments or vaccines available for MVD, making the control of the virus challenging.^7,11,17^

 The first Marburg case was reported on July 7, 2022, amidst simultaneous outbreaks of monkeypox and the COVID-19 pandemic in Ghana.^18^ Given the recent rise in reports of confirmed and suspected cases of MVD across various settings, this study aims to provide a comprehensive description and outline of the first-ever identification of MVD cases in Ghana. Additionally, it focuses on the country’s preparedness efforts and the public health actions taken in response to these outbreaks. By documenting these occurrences and analyzing the preparedness and response measures, valuable insights can be gained to enhance our understanding of MVD and inform future strategies to respond to these deadly diseases.

## Characterization of outbreaks recorded in Ghana

###  COVID-19 

 A day after the WHO announced COVID-19 as a pandemic, Ghana confirmed its first two infections of the virus.^19^ These initial cases were imported into the country by individuals who had recently travelled to Turkey and Norway and met the case definition for COVID-19 upon their return. SARS-CoV-2 virus confirmed in their samples by a PCR testing.^20,21^ In response, Ghana intensified surveillance at points of entry and expanded routine surveillance activities.^22-24^

 To combat the COVID-19 pandemic, Ghana adopted the “3Ts” strategy, which involves testing of samples, tracing of contacts, and treating confirmed cases.^25,26^ This approach ensures that individuals who meet the case definition are tested for the virus. Contact tracing is conducted to find and monitor persons exposed to confirmed cases, aiming to break the chain of transmission and detect cases early.^27,28^ Confirmed cases were treated at designated healthcare facilities to prevent severe or critical illness.

**Figure 1:**
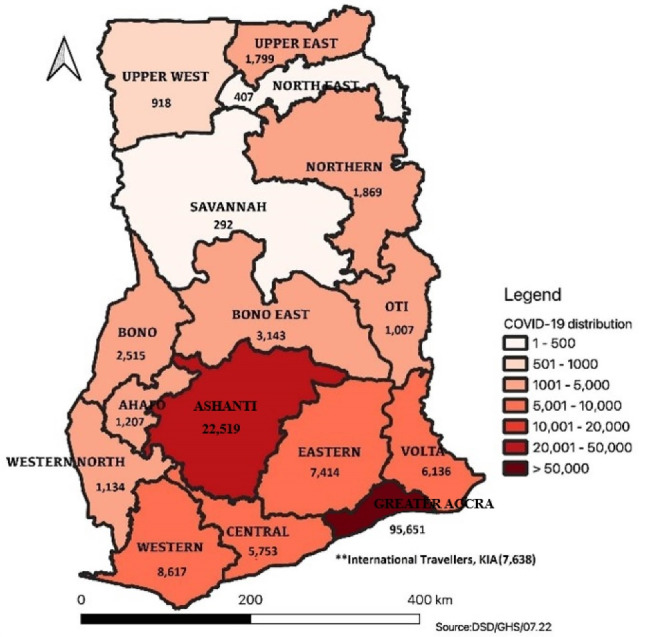
Regional breakdown of COVID-19 cases in Ghana, 24 July 2022

 As of July 24, 2022, Ghana has recorded a total of 168 019 cumulative cases of COVID-19, with 1457 deaths^29^ (See Figure 1). The current positivity rate stands at 6.8%, and all 16 regions of the country have reported confirmed cases. A total of 2 488 748 samples have been tested since March 2020, with 1 115 998 samples obtained through enhanced contact tracing efforts. Furthermore, Ghana has administered 18 396 070 doses of COVID-19 vaccines as of July 20, 2022, as part of its public health efforts in curbing the virus transmission.^29^

 The statistics demonstrated the ongoing efforts of Ghana to control the COVID-19 pandemic through robust testing, contact tracing, treatment, and vaccination campaigns.^30^ By implementing these public health actions, Ghana aimed to mitigate the impact of the virus and safeguard the health and safety of its people.^31^

###  Monkeypox virus

**Figure 2:**
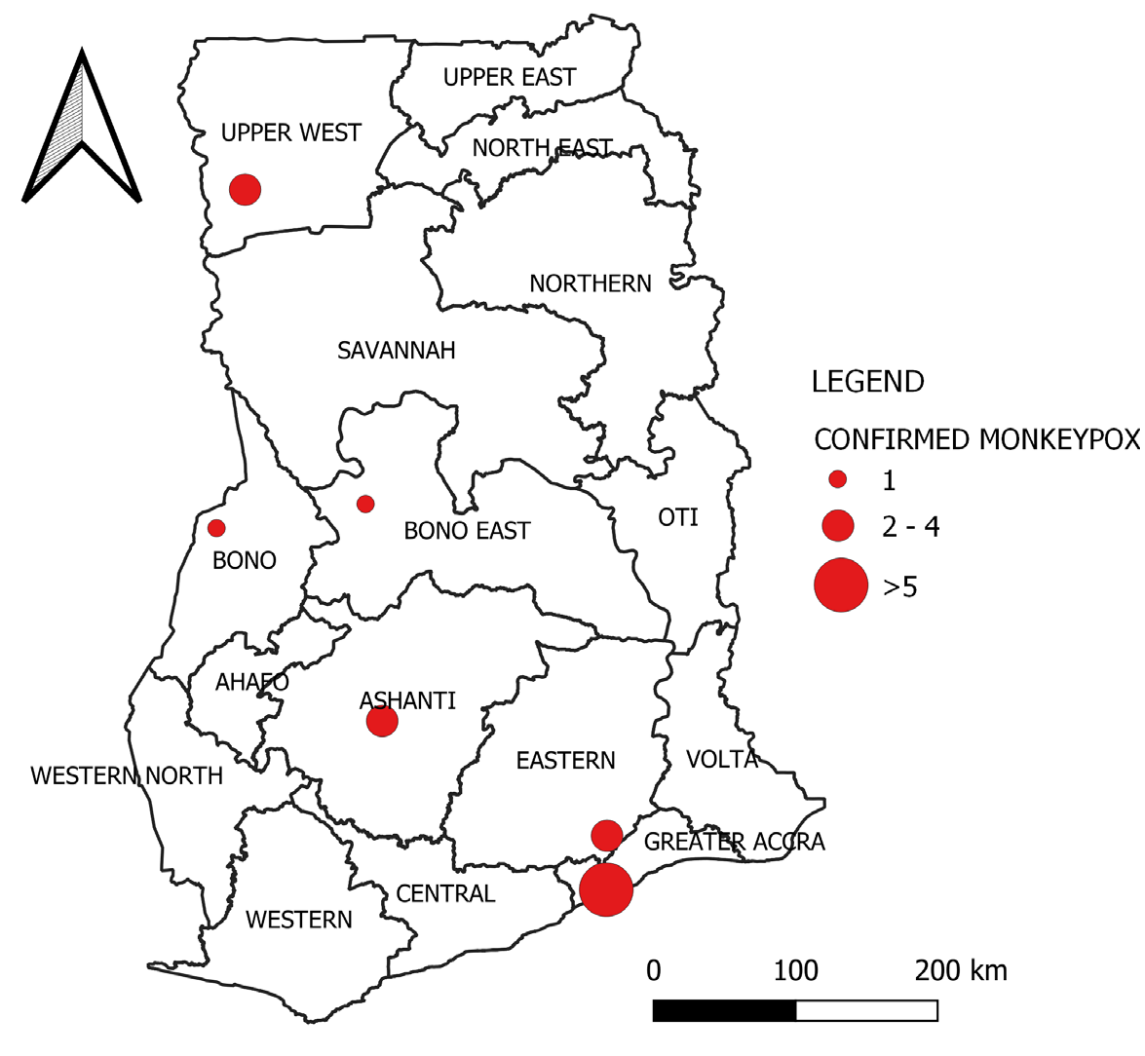
Distribution of confirmed human monkeypox virus in Ghana as of 15^th^ July 2022

 Monkeypox is a disease that occurs regularly and persistently in some African countries,^32,33^ but until July 8, 2022, Ghana had not reported any confirmed cases of Monkeypox in humans. However, the Ghana Health Service announced the first confirmed cases of Monkeypox on July 8, 2022. Following the announcement of the Monkeypox as a “Public Health Emergency of International Concern (PHEIC)”,^34^ surveillance efforts in Ghana have been intensified.^35^ As of July 15, 2022, Ghana has confirmed 33 Monkeypox virus cases^36^ (See Figure 2).

 The age of the people who had Monkeypox in Ghana varied from 9 months to 45 years old, and a higher proportion of males among the cases. Fortunately, no fatalities have been reported as a result of the virus.^37^ The cases have been detected in six out of the country’s 16 administrative regions. The areas affected included Eastern region, Ashanti region, Bono region, and the Bono East region located in the middle zone of the country and the Greater Accra region and the Upper West region located in the Southern and the Northern zone respectively. Notably, over 50% of the recorded cases that have been reported from Greater Accra, where the national capital is located.^38^

 In response to the Monkeypox outbreak, Ghana has implemented a comprehensive set of outbreak investigation and response measures in the affected districts of the regions.^39^ The initiation of contact tracing for confirmed cases was underway, accompanied by the collection of samples for laboratory testing. Strengthened surveillance activities have been prioritized to ensure an effective response to the outbreak,^40^ with a particular focus on enhancing the capacity of regional and district rapid response teams. These efforts aim to swiftly identify and contain the spread of Monkeypox, safeguarding public health within the affected areas.^41^ The primary objective of these measures is to exert control over the spread of Monkeypox in Ghana while minimizing its impact on public health. Through meticulous surveillance efforts, the implementation of comprehensive outbreak response strategies, and the reinforcement of response teams’ capabilities, Ghana proactively undertook measures to effectively manage the Monkeypox outbreak and safeguard the population against the continued transmission of the virus.

###  Marburg virus disease

**Figure 3:**
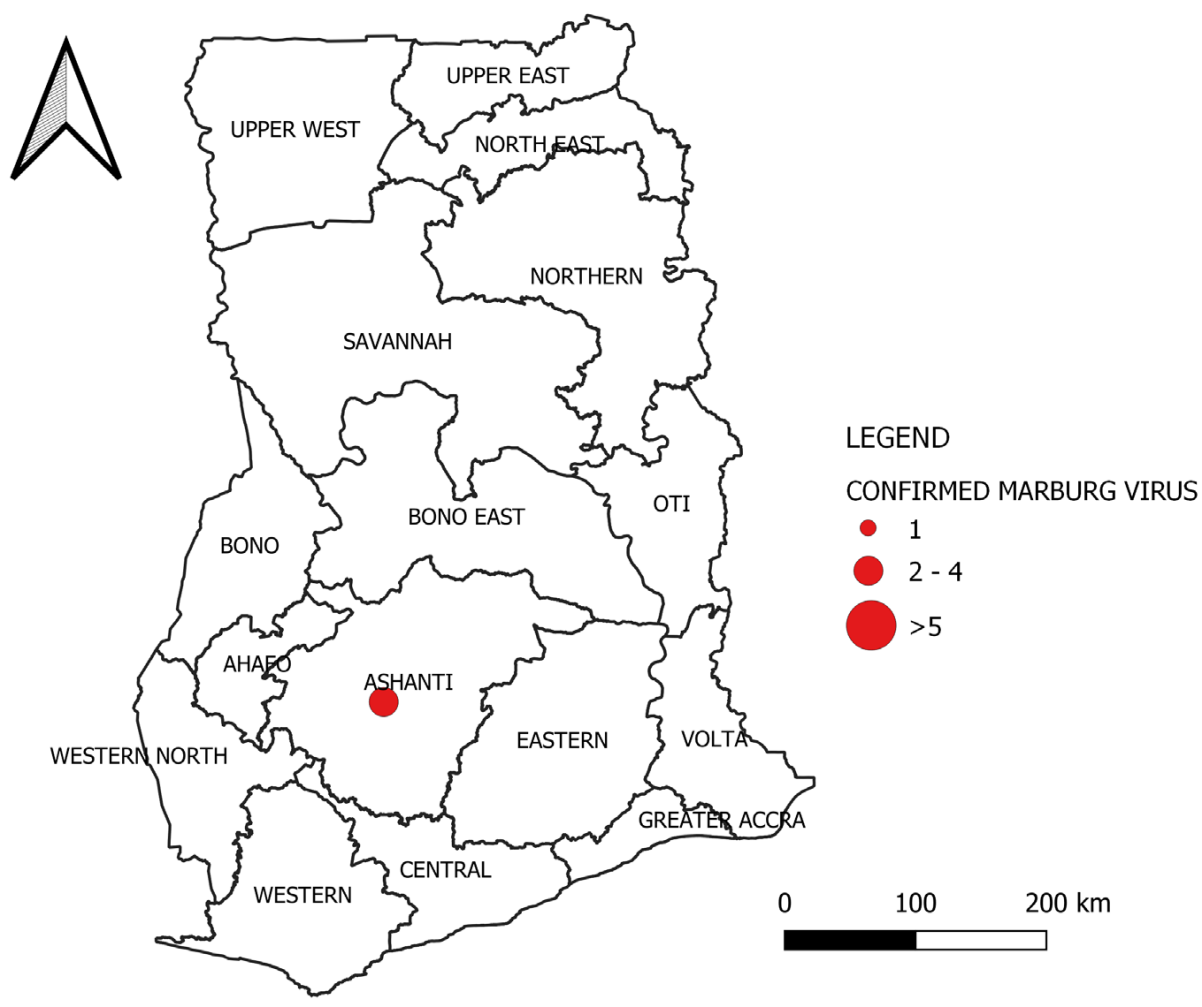
Distribution of confirmed Marburg virus in Ghana as of 24^th^ July 2022

 The Ghana Health Service (GHS) reported the country’s first 2 confirmed Marburg cases on 7^th^ July, 2022.^3,42^ The cases were identified in the Adansi North district of the Ashanti region. As of 24^th^ July, 2022, Ghana has recorded a total of three confirmed cases of Marburg, with a fatality rate of 66.7%, resulting in two deaths. The confirmed cases include two males and one female, all reported from the Ashanti region^16^ (See Figure 3).

 The index case was a man aged 26 years who presented symptoms of bleeding from the mouth and nostrils on June 22, 2022. Unfortunately, the patient died within 28 hours of admission to the hospital. A boy aged 1-year and 2-months, who was the first case’s offspring, was the second person to have the disease. The boy exhibited signs of Marburg disease and succumbed to the illness three days after admission to a healthcare facility in the Ashanti region.^3,16,42,43^

 A woman aged 24 years, who is married to the first case, was the third case of the disease. She tested positive for the Marburg virus after being identified as a contact of the confirmed case. Since July 26, 2022, she has been under isolation and receiving appropriate medical care.^3^

 The GHS has been actively conducting contact tracing and follow-up. A total of 118 contacts of the index case were identified and monitored. Among the immediate contacts, two individuals tested positive for the virus. The initial contacts, including the index case’s contacts, completed their mandatory 21-day follow-up period as advised by the WHO.^3^ Additionally, an additional 81 contacts have been identified from the second and third cases and are currently being followed up for any signs or symptoms of MVD.^3,16^

 These surveillance and response efforts aimed to identify and manage potential transmission chains, mitigate the transmission of the Marburg disease, and protect the health of the people in Ghana. The effort to monitor contacts and promptly isolate confirmed cases was essential strategies for containing the outbreak and preventing further transmission.^44,45^

## Preparation towards the outbreak of Marburg virus disease

 WHO mobilized a team of experts to assist Ghana’s health authorities in strengthening disease surveillance and response plans in light of the Marburg virus outbreak.^16,46^ The WHO experts collaborated with Ghana’s health authorities to establish a sensitive case definition for MVD, implement early testing and isolation protocols for confirmed cases, conduct comprehensive contact tracing efforts, ensure effective case management and isolation, and engage with communities to raise awareness about the risks and hazards associated with the disease.^16^

 To support the management of the outbreak, the WHO provided essential resources such as personal protective equipment (PPE) and other necessary logistics. Additionally, training opportunities were offered to healthcare staff to enhance their capacity in dealing with viral haemorrhagic fevers, including MVD. These training programs were conducted in collaboration with national and sub-national emergency response teams in Ghana.

 These preparations and collaborations were being implemented in a timely manner as ongoing investigations into the outbreak continue. The GHS has already initiated an investigation into the confirmed cases, and a comprehensive list of contacts of the confirmed cases is being compiled for further public health interventions and actions.

 The combined efforts of the GHS and the support from the WHO helped to strengthen Ghana’s response capabilities, mitigate the effect of the Marburg virus, and protecting the health and well-being of the population.^47,48^

## Lessons from COVID-19, monkeypox virus and MVD in Ghana for future outbreaks

 Lessons from the recent outbreaks provide valuable insights into the management of outbreak infectious diseases in the future. These outbreaks, especially COVID-19 have resulted in significant human mortality, associated morbidities, and strain on sociocultural and healthcare systems, as containment measures require substantial resources, including financial support, laboratory testing equipment and reagents, and skilled healthcare personnel.^49-51^ Ghana is among the few countries in sub-Saharan Africa simultaneously battling three outbreaks, posing challenges for effective containment of these infectious diseases.^52^

 In Ghana, COVID-19 vaccines have played an important role in lowering mortality and morbidity, but vaccine hesitancy remains a concern.^53^ A study conducted among the adult population revealed that 21% were unwilling to take the vaccine, and an additional 28% remained undecided about vaccination.^54^ Overcoming vaccine hesitancy will be a challenge as health officials assess the risk of monkeypox infection and consider introducing vaccination.^55^

 In its response to the pandemic, Ghana implemented proactive measures, including updating its emergency preparedness plan and establishing emergency operation centers in each region.^56^ These strategies have significantly contributed to the control of all ongoing outbreaks in Ghana.^24^ The country possesses testing capacity for highly infectious diseases, including MVD, at institutions such as the “Noguchi Memorial Institute for Medical Research (NMIMR)”, the “National Public Health and Reference Laboratory”, and the “Kumasi Centre for Collaborative Research (KCCR)”.^57^ During the initial COVID-19 response and the monkeypox outbreak, these laboratories served as the primary testing facilities. With the emergence of MVD, Ghana will need to enhance the testing capacity of these laboratories and regional “Public Health and Reference Laboratories” to confirm cases that meet the defined criteria for MVDs.

 Contact tracing, a vital strategy employed during the COVID-19 pandemic and the human monkeypox outbreak, has been initiated following the confirmation of MVD cases.^58,59^ The Ashanti Regional Health Directorate of GHS compiled a comprehensive list of contacts from the three confirmed cases, and conducted an active follow-up while individuals are placed under quarantine. These efforts aim to prevent further transmission and facilitate prompt intervention measures.

 Overall, Ghana’s experience with COVID-19 pandemic and monkeypox virus outbreak highlighted the importance of proactive preparedness, effective laboratory testing capabilities, and comprehensive contact tracing in combating infectious disease outbreaks. Applying these lessons to the management of Marburg virus will be critical in minimizing the impact of the ongoing outbreak and protecting public health in Ghana.

## Recommendations for responding to future outbreaks

###  Health services and partners

 To improve public health responses to outbreaks, beginning with their core functions,^60^ Ghana needs to upgrade its surveillance systems so that it can identify and track potential MVD cases effectively. This can be achieved through the implementation of robust surveillance mechanisms, such as real-time data collection, analysis, and reporting systems.^61^ Integration of advanced technologies, including genomic sequencing and rapid diagnostic tests, can significantly improve the accuracy and speed of case detection and response.^62,63^ Moreover, using and harmonizing digital surveillance tools in the country can improve the quality and efficiency of surveillance and case finding.

 Second, Ghana should develop and strengthen its response strategies for future outbreaks, building upon the lessons learned from previous outbreaks such as COVID-19 and monkeypox.^64^ This can involve establishing dedicated emergency response teams with clear roles and responsibilities, as well as coordination structures that facilitate efficient communication and collaboration among different stakeholders. Efficiently assuring the quick deployment of critical resources, such as health workers, supplies, and equipment, is critical for effectively controlling and managing epidemics.^65^

 Third, effective risk communication and community engagement are essential in controlling outbreaks. Ghana should invest in tailored risk communication strategies that target various population groups and consider the local context. Providing clear and accurate information about MVDs, its transmission dynamics, prevention measures, and the importance of community engagement is crucial. Media campaigns, community meetings, and engagement with community leaders, healthcare providers, and other influential individuals can be effective channels for communication.

 Fourth, Ghana’s healthcare system should be adequately prepared to handle potential cases of MVD. This requires ensuring that healthcare workers are trained on proper case management protocols, infection prevention and control procedures.^66^ Also, the proper utilization of PPE plays a crucial role in effectively managing infectious diseases.^67^ Additionally, maintaining an adequate and continuous supply of medical equipment, diagnostic tools, therapeutics, and vaccines is vital.^50^ It is important to establish or identify isolation facilities in advance to promptly isolate and treat suspected and confirmed cases while minimizing the risk of transmission within healthcare settings.

 Finally, collaboration with local and international partners is crucial for advancing knowledge about MVD and improving outbreak response. Ghana should invest in research efforts focused on understanding the viruses’ epidemiology, transmission dynamics, host reservoirs, and potential treatment options. Collaborative research initiatives can facilitate knowledge-sharing, capacity-building, and resource mobilization.^68^ Furthermore, participating in global networks and sharing of data and biological samples can contribute to a broader understanding of MVD and its control.^69^

###  Policy makers

 Policymakers play a crucial role in safeguarding public health and creating a healthy community,^70^ especially in preparing for and responding to future outbreaks. With a focus on the current outbreaks of COVID-19, monkeypox, and Marburg disease in Ghana, policymakers can prioritize public health issues and employ mechanisms and plans from “the Ottawa Charter for health promotion”^71^ to effectively address and prevent potential outbreaks. Policymakers have a significant responsibility in identifying and prioritizing public health issues to ensure a healthy community.^72^ In the context of preparing for future outbreaks, they need to assess the risks posed by various infectious diseases and potential epidemics. By analyzing data, consulting with experts, and involving communities, policymakers can identify critical health threats and allocate resources accordingly.

 The Ottawa Charter for health promotion offers essential strategies that policymakers can integrate into their policymaking process to strengthen preparedness and response for future outbreaks^71^;

####  a. Building healthy public policies

 Developing and implementing policies that support disease surveillance and reporting, establishing comprehensive emergency response plans, and ensure sufficient funding for healthcare infrastructure is vital in outbreak response as a country. By building a strong framework of public health policies, policymakers set the foundation for effective outbreak prevention and management.^73^

 Although the passing of the Public Health Act, 2012 (Act 851), represented a significant step in our public health plans, it became evident during the COVID-19 pandemic response that the Act had several deficiencies. In addressing the gaps in the Act during the response, policymakers signed several executive instruments, including the Executive Instrument (EI) 68 of 2020, EI 164, and a new Act known as the Imposition of Restrictions Act 2020 (Act 1012),^56^ to supplement the Public Health Act. However, recognizing the growing threat of future outbreaks, the Public Health Act require immediate amendment to address these weaknesses and improve its effectiveness in combating emerging health crises.

 In addition to amending the Public Health Act, there is a pressing need to strengthen the country’s public health response while also establishing a dedicated funding source through the Act, specifically allocated for addressing public health threats in the future. Allocating earmarked funds annually into a dedicated fund purposed for future outbreaks becomes essential. This measure ensures that funding uncertainties during outbreaks are minimized, protecting funding for other vital sectors of the economy. The commendable passage of the national vaccine institute bill reflects the government’s commitment to advancing vaccine development. Nevertheless, to expedite the production of vaccines in-country, additional actions must be taken. This may include forming strategic partnerships with relevant stakeholders, investing in research and development, and providing necessary resources and infrastructure to support local vaccine production.^74^

 Lastly, it is imperative for policymakers in Ghana to expeditiously establish a dedicated Public Health Council to regulate all public health practices in the country. Given the growing importance of public health within our healthcare system, this sector requires serious attention and a centralized body to oversee its operations. By establishing a Public Health Council, crucial gaps and challenges related to public health human resources, training, remuneration, and other significant factors can be effectively addressed. This proactive approach will considerably strengthen the public health sector’s preparedness and response capabilities ahead of future outbreaks.

 Policymakers have the ability to shape public health priorities through the formulation of policies that prioritize disease surveillance, emergency response plans, and adequate healthcare infrastructure. While acknowledging the efforts made with the Public Health Act and the national vaccine institute bill, there is an urgency to address identified shortcomings, enhance public health response, and secure dedicated funding for future outbreaks.

####  b. Creating supportive environments

 Establishing essential infrastructures that support the well-being of a healthy community is what creating supportive environments involves. Policymakers have the key to advancing in this area by allocating investments towards healthcare facilities, laboratories, and effective medical supply chains, thereby boosting the overall capacity for detecting and responding to outbreaks promptly. Also, their work to ensure safe housing, clean water, and proper sanitation is very important in preventing disease spread and making communities more resilient during outbreaks.^73^

 The Agenda 111 initiative is a praiseworthy step in this direction, concentrating on the construction of 111 hospital projects across Ghana. As of August 2021, promising progress has been achieved, with 88 sites starting construction.^75^ To further improve outbreak response capabilities, policymakers should give priority to the establishment of infectious diseases centers in the Ashanti region and the Northern region, serving the middle and northern belts of the country. This strategic move will considerably strengthen the country’s preparedness in addressing infectious diseases in these regions.

 Moreover, the long-anticipated setting up of the Ghana CDC holds great potential in tackling diseases head-on.^76^ This dedicated center would play a vital role in coordinating and leading the country’s response to outbreaks and public health emergencies, unifying efforts, and fostering a cohesive approach. The Ghana CDC will play a complementary role alongside the existing bodies of the GHS in combating future outbreaks.

 By investing in these healthcare infrastructures and disease control measures, policymakers can create an environment that not only enables effective outbreak response but also strengthens the overall healthcare system to cater to diverse health needs. The progress made with Agenda 111 and the potential establishment of the Ghana CDC are significant milestones towards achieving these goals.^77^

####  c. Strengthening community action

 By fostering cooperation among local organizations, community leaders, and healthcare providers, policymakers can access the community’s resources and expertise. This approach involves implementing community-based surveillance, setting up early warning systems, and supporting community-driven initiatives for infection prevention and control.^78^ To improve community engagement in outbreak preparedness, the Information Service Division (ISD) of the Ministry of Information should communicate regularly with the Health Promotion division of the GHS. This collaboration will ensure consistent sharing of information about potential disease outbreaks with the public, shifting from reactive approaches to proactive information dissemination. Furthermore, engaging local authorities in health promotion meetings for the communities they serve will cultivate a sense of ownership and responsibility for the people’s health.^79^ To effectively enable local communities in addressing health issues and potential outbreaks, it is vital to allocate resources from the government and partner organizations. These resources will enable community-led responses and establish suitable local communication channels to distribute vital health information during potential outbreak threats. Policymakers must actively involve the community in outbreak preparedness efforts by enhancing collaboration and leveraging local expertise. Proactive information sharing, involvement of local authorities, and the allocation of resources will enable communities to control their own health and help authorities respond to potential outbreaks efficiently.^78^

####  d. Developing personal skills

 Enhancing health literacy and knowledge is a key step in enabling individuals to protect themselves and their communities during outbreaks.^80^ Policymakers have a crucial role in backing educational campaigns that inform people about infectious diseases, preventive measures, and early symptoms, allowing people to take proactive actions in effectively managing outbreaks. To strengthen the response to future outbreaks, it is vital for governments and individuals responsible for public health to concentrate on the training and retraining of health personnel.

 To accomplish this, policymakers should provide direct incentives for health workers based on merit rather than subjective choices. They should also ensure easy access to scholarships and workshops for health professionals, enabling them to pursue training opportunities, thus cultivating skilled and willing health workers. Furthermore, they should secure fair distribution of opportunities for further education and ongoing training, with the Ministry of Health and relevant agencies taking steps to support the educational goals of health staff.

 One urgent concern to tackle is the fair compensation of health personnel, as insufficient salaries can lead to brain drain, creating significant difficulties for future outbreak response in Ghana and throughout the sub-Saharan region as well. This pressing issue demands immediate attention and should initiate a national dialogue to devise effective solutions.

 A study in Ghana found that the work performance of health workers was influenced by intrinsic factors such as how satisfied they were with their jobs, how well they were provided with logistics, and how conducive their work environment was, as well as extrinsic factors including financial rewards, housing, and transport.^81^ Therefore, addressing these motivational factors is essential in ensuring the commitment and effectiveness of healthcare workers in responding to outbreaks.^82^

####  e. Reorienting health services

 To prepare for future outbreaks, policymakers can prioritize strengthening primary healthcare and preventive services. By focusing health services on early detection, effective contact tracing, and extensive vaccination campaigns, policymakers can ensure prompt interventions, limit transmission, and enhance the overall robustness of the public health system. In the past, government spending on health has mainly gone to clinical care^83^; however, the paradigm is now changing towards a more economical and sustainable approach through preventive health measures. The need to shift the healthcare system towards public health is becoming more obvious, as many countries are acknowledging its importance in protecting population health and wellbeing for the long term.^84^

 These recommendations are aimed at strengthening Ghana’s preparedness and response capacities for epidemic prone diseases, based on lessons learned from previous outbreaks and scientific evidence. Continued evaluation, monitoring, and adaptation of strategies are essential to address the evolving nature of infectious disease threats. As each disease has its own unique characteristics, the lessons learned from COVID-19, monkeypox, and Marburg outbreaks may not directly apply to future outbreaks. Therefore, continuous monitoring of the global and regional public health landscape and consultation with relevant experts would be essential in shaping preparedness and response strategies for responding to future outbreaks in Ghana.

## Conclusion

 The concurrent occurrence of COVID-19, human monkeypox virus, and MVD outbreaks imposed significant strain on Ghana’s health system in recent months. However, the system has demonstrated resilience in effectively managing and responding to these outbreaks. Moving forward, it is imperative to prioritize a coordinated global effort that emphasizes robust surveillance systems and rapid case detection. These measures are crucial for containing outbreaks and preventing further spread of infectious diseases.

 The experience gained from the COVID-19 response, human monkeypox outbreak, the MVD outbreak underscores the importance of a well-coordinated global response. By prioritizing surveillance, rapid case detection, and effective outbreak containment strategies, Ghana and the international community can mitigate the burden of future infectious disease outbreaks and protect public health. Policymakers must also consider the strategies outlined in this paper, with reference to the Ottawa Charter for Health Promotion, to ensure that policies are directed towards a successful response to future outbreaks with a special focus on building healthy public policies and developing personal skills of health personnel across the country.

## Acknowledgements

 We thank the Office of the President, Ghana; the Health Ministry, the Ghana Health Service; WHO (Ghana country office); GFELTP and relevant stakeholders for their contribution in the outbreak response in Ghana.

## Competing Interests

 The authors stated that they have no conflicts of interest.

## Ethical Approval

 Ethical approval was not required for this study.

## Funding

 The research did not receive any funding from outside sources.
